# Repair of Acute Type-A Aortic Dissection in the Present Era: Outcomes and Controversies

**DOI:** 10.1055/s-0039-3401810

**Published:** 2020-04-09

**Authors:** Ellie Moeller, Marcos Nores, Sotiris C. Stamou

**Affiliations:** 1 Department of Cardiothoracic Surgery, JFK Medical Center, Atlantis, FL

**Keywords:** aortic dissection, outcomes, circulatory arrest, pathophysiology, adults

## Abstract

Acute Type-A aortic dissection (AAAD) remains a surgical emergency with a relatively high operative mortality despite advances in medical and surgical management over the past three decades. In spite of the severity of disease, there is a paucity of studies reviewing key controversies surrounding AAAD repair and management. A systematic literature search was performed using Cochrane review and PubMed bibliography review. Abstracts were first reviewed for general pertinence and then articles were reviewed in full. Literature review indicates that use of moderate hypothermia and antegrade cerebral perfusion is a safe alternative to deep hypothermia. In hemodynamically stable patients, axillary cannulation may be substituted for femoral cannulation. With regard to the technical aspects of repair, preserving the aortic root whenever possible and performing the distal anastomosis with the open distal technique rather than with the clamp on is the preferred approach. In patients with a patent false lumen, close monitoring is indicated. As demonstrated by the literature, significant improvement of early and late mortality over the past years has occurred in patients presenting with AAAD. Repair of acute Type-A aortic dissection remains a challenge with high operative mortality; however, improvement of surgical techniques and management have resulted in improvement of early and late clinical outcomes.

## Introduction

Acute Type-A aortic dissection (AAAD) remains a surgical emergency with a relatively high operative mortality despite advances in medical and surgical management over the past three decades. 1 2 3 Due to the prevalence and severity of the disease process, significant research exists surrounding the optimal management and expected outcomes of AAAD. 4

However, underlying the extensive studies on this topic have several controversies regarding key topics of management. Specifically, topics including deep versus moderate hypothermia, cannulation site, technical aspects of repair, hemodynamic instability, and the fate of the false lumen are debated. There is a paucity of studies investigating the current status of these key topics. This review is necessary to explore and understand controversies surrounding AAAD management.

## Methods

A literature review was conducted using PubMed and. We aimed to review all studies on repair of Type-A aortic dissection using PubMed and Cochrane Library databases. Abstracts were first reviewed for general pertinence, and then articles were reviewed in full. Additional literature search was performed by reviewing the reference lists of articles. Our search process concluded in October 2018.

## Results

### Deep Hypothermia versus Moderate Hypothermia

Deep hypothermic circulatory arrest (DHCA) is considered by many experts to be the standard of care for surgical repair of AAAD. 5 6 Deep hypothermia decreases brain metabolism by approximately 50% per 6°C drop in organ temperature and enables full neurologic recovery after the interval of interruption in brain perfusion. 7 8 However, due to prolonged duration of cardiopulmonary bypass (CPB) typically associated with the profound hypothermia induced by DHCA, increased complication rates including postoperative bleeding, endothelial dysfunction, neuronal apoptosis, and postoperative pulmonary complications are reported to exist. 5 In addition, DHCA induces vasoconstriction and decreases regional cerebral blood flow. 5 The combination of complications and unfavorable physiologic changes associated with DHCA have led to a gradual shift toward using moderate degrees of hypothermia (MH). 5

Recent studies have found MH to be independently associated with lower risk of mortality and major adverse cardiac and cardiovascular events during AAAD repair. 5 9 A retrospective cohort study by Algarni et al 5 of 128 patients compared the two strategies of cooling (DHCA, <20°C; and MH, 22–28°C) to repair AAAD at a single center, and their results are shown in Table 1 . Algarni et al 5 reported significantly higher rates of stroke with persistent neurologic deficit (21 and 13%, *p* = 0.042) and low cardiac output syndrome (26 and 5%, *p* < 0.001) in the DHCA group compared with the MH group, respectively. Mortality was almost two-fold higher in the DHCA group than the MH group (28 and 16%, *p* = 0.07). 5 However, in addition to these findings, CPB time and blood transfusion were significantly higher in the DHCA group than the MH group ( *p* = 0.04).

**Table 1:** Summary of study findings on hypothermia

Study	Patient	Finding
Algarni et al 5	Risk of stroke, low cardiac output syndrome, and mortality between medium and deep hypothermia	Significantly higher rates of stroke with persistent neurologic deficit (21 and 13%, *p* = 0.042) and low cardiac output syndrome (26 and 5%, *p* < 0.001) with profound hypothermia compared with moderate. Mortality was almost two-fold higher in the profound group than the moderate group (28 and 16%, *p* = 0.07)
Stamou et al 10	Survival rates of 324 patients undergoing AAAD repair with either DHCA, retrograde or anterograde cerebral perfusion	No significance between types of cerebral protection used. Predictors of operative mortality were hemodynamic instability and CPB time
Bakhtiary et al 12	Clinical results of 120 patients undergoing AAAD repair with mild systemic hypothermia	Permanent neurologic deficits were seen in 4.2% of patients. The 30-day mortality rate was 5%. Follow-up of 2.8 years showed a survival rate of 87%

Abbreviations: AAAD, acute Type- A aortic dissection; CPB, cardiopulmonary bypass; DHCA, deep hypothermic circulatory arrest.

These findings raise questions as to whether hypothermic temperature may also play a confounding role, as supported by a recent study by the senior author. 10 We compared survival between 324 patients undergoing AAAD repair with either DHCA, retrograde, or anterograde cerebral perfusion. 10 Using multivariable logistic regression, we found that independent predictors of operative mortality were hemodynamic instability and CPB time, not type of cerebral protection used. 10 The strongest negative effect of DHCA originates from increased CPB times and subsequent length of operation in comparison with MH. 11 12 13 Extended CPB times during cardiac surgery are implicated in increased risk of acute renal insufficiency, stroke, and mortality. 13 14 15 These effects can be compounded based on the condition of the patient. Diminished hematocrit and glycemic levels can increase perioperative risk during the use of CPB. 16 17 In our study, the median CPB time was 219 minutes for the DHCA group and 173.5 minutes for the MH group ( *p* < 0.001). 13 Also, the number of patients reaching the extended CPB time of 240 minutes in the DHCA group tripled that of the MH group ( *p* < 0.015). An increased prevalence of postoperative risk found using DHCA might actually arise secondary to increase cardiopulmonary bypass times. 13 However, limitations of moderate hypothermia may include higher risk of injury to the distal organs secondary to warmer temperatures, especially if body arrest time is prolonged. Further, patients who need more complex repairs, such as total aortic arch replacement may be better served by DHCA, or moderate hypothermia with dual perfusion of the brain via axillary artery and the body via the femoral artery.

Although additional studies are needed to investigate this controversy, MH with selective antegrade cerebral perfusion seems to be a safe strategy that accomplishes excellent outcomes with relatively low rate of neurologic complications and lower CPB times compared with DHCA. Both techniques, however, should aim toward limiting cardiopulmonary bypass times with efficient planning of the operative steps, such as completing the aortic root repair while cooling the patient to hypothermia.

### Axillary versus Femoral Cannulation

Considerable debate remains regarding the optimal cannulation site in patients undergoing AAAD repair, specifically comparing clinical outcomes of axillary artery cannulation (AXC) with femoral artery cannulation (FAC). 18 19 20 21 22 Concern of flow reversal in the thoracoabdominal aorta with FAC exists and has contributed to a trend of using the AXC site for CPB. 23 Recent studies focus on early and late outcomes of AXC versus FAC and inform the year-old controversy. 18 20

A meta-analysis by Ren et al reviewed nine nonrandomized studies comparing outcomes in patients undergoing AAAD repair with AXC or FAC, with results shown in Table 2 . 18 Fixed-effect modeling showed significantly lower incidence in short-term mortality (odds ratio [OR] = 0.25; 95% confidence interval [CI]: 0.15–0.42; *p* < 0.01) and in neurologic dysfunction (OR = 0.46; 95% CI: 0.29, 0.72; *p* < 0.01) in the AXC group. A meta-analysis by Benedetto et al, 23 composed largely of retrospective studies, showed central cannulation, including the axillary artery, to be superior to peripheral cannulation of the femoral artery in the short term, as shown in Table 2 . These findings are hypothesized to be due to flow reversal in the thoracoabdominal aorta with femoral artery cannulation, increasing risk of brain or organ malperfusion. 19 24 However, a recent study of 215 patients by Klotz et al 25 found no significant differences in postoperative neurologic deficits ( *p* = 0.449) or 30-day mortality ( *p* = 0.699) between patients undergoing central and femoral cannulation. Despite this, most literature trends in favor of axillary cannulation, and most surgeons have adopted an antegrade perfusion strategy with axillary artery cannulation. 26

**Table 2:** Meta-analysis results of axillary versus femoral cannulation

Outcome	Odds ratio	Relative risk	95% confidence interval	*p* -Value
*Ren et al* 18 :				
Short-term mortality	0.25	–	0.15–0.42	0.01
Neurologic dysfunction	0.46		0.29–0.72	0.01
Malperfusion incidence	0.84	–	0.37–1.90	0.67
*Benedetto et al* 23 :				
In-hospital mortality	–	0.59	0.48–0.7	<0.01
Permanent neurologic deficit	–	0.71	0.55–0.9	0.005

Note: odds ratios and relative risk are shown as comparison of axillary/central artery cannulation versus femoral/peripheral artery cannulation.

With regard to long-term survival, data are limited. However, a retrospective study of 305 patients showed comparable 5-year survival between the axillary and femoral cannulation ( *p* = 0.52). 20 Cox's regression analysis demonstrated predictors of long-term mortality to be age ( *p* < 0.001), stroke ( *p* < 0.001), prolonged CPB time ( *p* = 0.001), hemodynamic instability ( *p* = 0.002), and renal failure ( *p* = 0.001). 20 Additional studies demonstrated similar findings, presenting evidence that repair with AXC reduces overall mortality and neurologic complications when compared with FAC. 27 28 29 30 31 These findings are summarized in Table 3 .

**Table 3:** Summary of study findings on cannulation site

Study	Patient	Finding
Stamou et al 20	5-year survival in patients undergoing AXC vs. FAC	No difference in 5-year survival between groups undergoing AXC versus FAC
Moizumi et al 27	Pre- and postoperative predictors of hospital death in patients with AAAD	Vischeral ischemia (OR = 18.4, *p* = 0.0028) and absence of axillary artery perfusion (OR = 8.2, *p* = 0.0014) were independent preoperative and operative predictors of hospital death
Reuthebuch et al 28	Clinical and neurological outcomes of patients undergoing subclavian artery cannulation versus femoral artery cannulation	Significantly improved neurological outcome ( *p* = 0.0057), decreased postoperative bleeding ( *p* < 0.0001), decreased incidence of MI ( *p* < 0.0001), and decreased 30-day mortality ( *p* = 0.0179) in patients undergoing subclavian artery cannulation compared with FAC
Pasic et al 30	Neurological complications and hospital mortality in patients undergoing AAAD repair with AXC versus FAC	Postoperative complications occurred in both groups, at nonsignificantly higher rates in FAC compared with AXC
Etz et al 31	Mortality and stroke in patients undergoing AAAD repair with AXC versus FAC	AXC had significantly better outcomes than FAC ( *p* = 0.02)
Benedetto et al 23	Meta-analysis of 4,476 patients comparing central and peripheral cannulation in patients undergoing aortic surgery	Central cannulation (AXC) showed decreased in-hospital mortality (RR = 0.59, *p* < 0.001) and permanent neurological dysfunction (RR = 0.71, *p* = 0.005) when compared with peripheral cannulation (FAC)
Klotz et al 25	Postoperative cerebral infarction, dialysis, and 30-day mortality in patients undergoing AAAD repair with either AXC or FAC	Comparable postoperative cerebral infarction and 30-day mortality between the groups ( *p* = 0.699). Nonsignificantly higher rates of need for dialysis in patients undergoing FAC ( *p* = 0.073)

Abbreviations: AAAD, acute Type- A aortic dissection; AXC, axillary artery cannulation; FAC, femoral artery cannulation; OR, odds ratio; RR, risk ratio.

Debate is maintained through the studies that found no difference in survival or complication rates between AXC and FAC. 32 33 34 However, despite this, majority of the evidence demonstrates that perfusion through the AXC site may be clinically advantageous to FAC. Furthermore, these findings demonstrate that, regardless of cannulation strategy adopted, it is critical to carefully monitor procedures and respond adequately to adverse events. 26

### Direct Aortic Cannulation

This technique, which also avoids retrograde flow in the downstream aorta, is an alternative to time-consuming axillary artery access. The Hannover group reported their experience of direct aortic cannulation in 122 patients with aortic dissection. 35 Malperfusion occurred in three patients (2.5%). Hospital mortality was 15% for the entire cohort (18 patients). Permanent neurological dysfunction was detected in 15 patients (12%), whereas temporary neurological dysfunction occurred in 21 (17%). Total arch replacement was performed in 31 patients (25%).

### Technical Aspects of Repair

An additional area of controversy within AAAD repair is the different techniques of proximal and distal root reconstruction. Choice of reconstruction technique is largely based on viability and function of affected tissue; however, perioperative outcomes are poorly studied. 36 Importantly, surgeon preference may play a role in which technique is utilized, and it is therefore essential to fully understand the risk of each technique.

The most common surgical techniques for proximal root reconstruction include aortic valve (AV) resuspension for structurally normal valves and sinuses, aortic valve replacement (AVR) for a structurally abnormal valve but intact sinuses, and root replacement if both the valve and sinuses are abnormal. 36 A retrospective cohort study by Gunn et al 37 found that the actuarial 10-year survival rates were greatest in AV resuspension, followed by root replacement and AVR (72, 56, and 36%, respectively), and were significantly increased in patients who underwent AV resuspension as compared with AVR ( *p* = 0.011). This finding is consistent with the premise that increasingly compromised tissue predisposes to greater risk. Gunn et al also showed independent predictors of operative mortality to be hemodynamic instability (OR = 1.9; 95% CI: 0.03, 0.75; *p* = 0.021) and CPB time greater than 200 minutes (OR = 1.9; 95% CI: 0.04, 0.54; *p* = 0.004). Again, consistent with the extent of abnormalities, CPB was significantly longer in root replacement compared with AV resuspension ( *p* < 0.001) or AVR ( *p* = 0.027). 36

While most surgical repair focuses on proximal repairs as described previously, the dissection often propagates beyond the arch to the aortic bifurcation, described as a DeBakey-I dissection. Among proximal strategy repairs, most patients are left with a patent “Type-B” dissection, or false lumen, which yields a reoperation rate of more than 30% to address a dissecting aneurysm. To address this, standard proximal repair may be supplemented by thoracic stent-grafting through the open arch. A study by Pochettino et al 38 demonstrated that antegrade stent graft deployment in patients with DeBakey-I dissections obliterated the false lumen in 80% of patients. Furthermore, short-term results were comparable between the stented and nonstented groups, despite longer CPB times in the stented group. In patients with DeBakey-I dissections; therefore, consideration of antegrade stent grafting should be given to lower morbidity and mortality.

With regard to construction of the distal anastomosis, open distal anastomosis under circulatory arrest or distal aortic clamping with hypothermic cardiopulmonary bypass are the primary surgical approaches. Recent studies comparing techniques demonstrate comparable outcomes and survivals. 39 40 41 42 43 44 Although outcomes are similar, distal aortic clamping has been reported to distort the posterior tip of the clamp and does not allow resection of the injured clamping site, both of which may lead to higher reoperation for bleeding rates. 45 As such, open distal anastomosis under circulatory arrest is preferred technically. 46

### Hemodynamic Instability

Although previous studies related hemodynamic instability to differences in early and late outcomes following AAAD, no studies have previously quantified late survival between hemodynamically stable and unstable patients. 47 A recent study by Conway et al 48 was consistent with prior findings, as shown in Table 4 . This study demonstrated significantly higher rates of postoperative complications in patients with hemodynamic instability, including cardiac arrest ( *p* < 0.001), operative mortality ( *p* < 0.001), and acute renal failure ( *p* = 0.001). Late survival followed a similar trend, with decreased late survival among patients presenting with hemodynamic instability. At 1 year, 82% of hemodynamically stable patients and 57% of hemodynamically unstable patients were alive, and at 10 years, 63 and 44% patients, respectively. 48 From these studies, the authors found that excessive mortality occurs early in the postoperative course in patients presenting with hemodynamic instability. As such, treatment of these patients must be individualized.

**Table 4:** Summary of study findings on surgical era

Study	Patient	Finding
Fann et al 76	Surgical survival rates of patients with AAAD between 1963 and 1992	Earlier operative year, hypertension, cardiac tamponade, renal dysfunction, and older age were independent determinants of operative death.
Conway et al 77	Early postoperative outcomes and actuarial-free survival in patients undergoing AAAD repair between 2000 and 2005 and 2006 and 2010	Operative mortality was significantly higher in earlier surgical era (24% in 2000–2005, 12% in 2006–2010; *p* = 0.013). Earlier date of surgery, hemodynamic instability, and CPB >200 minutes were independent determinants of operative mortality

Abbreviations: AAAD, acute Type- A aortic dissection; CPB, cardiopulmonary bypass.

Malperfusion syndromes include cardiac, cerebral, renal, mesenteric, iliofemoral, innominate, and spinal and are associated with high hospital mortality and increased postoperative complications. 49 50 A study of 221 AAAD patients by Geirsson et al 51 found malperfusion in 26.7% of patients, with more than 30% of these patients experiencing two or more malperfusion syndromes. Cardiac ( *p* = 0.02) and cerebral malperfusions ( *p* < 0.001) were significant risk factors for in-hospital mortality, and cerebral malperfusion was a significant risk factor for decreased long-term survival ( *p* = 0.0002). Recommended treatment of malperfusion syndromes is rapid restoration of flow into the true lumen and obliteration of the false lumen to restore flow to all distal aortic branches.

In patients who are high risk for open repair, including those with significant comorbidities or anatomic challenges, endovascular treatment may provide an alternative. Although reports of endovascular repair are typically limited by small sample size, studies have shown promising outcomes for in-hospital and 30-day mortality rates. A study by Vallabhajosyula et al 52 demonstrated zero in-hospital and 30-day mortality in patients treated endovascularly who were prohibited from open repair due to hemodynamic instability, extreme frailty, malignancy, and severe fibrosis or osteomyelitis of the mediastinum. Similar studies reported comparable outcomes in small series. 53 54 55 56 These studies demonstrate that in patients who are hemodynamically unstable, have prohibitive comorbidities, or present anatomical challenges, endovascular repair of the ascending aorta is technically feasible. Although feasible in small series this technique is challenging and is presently not an acceptable treatment of Type-A aortic dissection.

### Fate of the False Lumen

Persistent patent false lumen in the aorta is common in AAAD and may be associated with poor long-term prognosis. 57 58 59 60 61 62 63 64 65 66 67 68 69 70 71 72

Analysis of the natural history of the residual aorta after AAAD repair provides insight into the outcomes for patients with persistent patent false lumen. The presence of a patent false lumen has been shown to be a significant risk factor for aortic enlargement, increasing the likelihood for reoperation. 73 These findings are consistent with those of Park et al, 62 who found that the primary indication for reoperation following AAAD repair was progressive enlargement of the false lumen, affecting 43% of patients. Enlarged aortic diameter has been shown to be an independent predictor for chronic dissection. 74 In addition to aortic enlargement, patent false lumen can lead to multiple reentries between the false and true lumen which requires reintervention, as described by Rylski et al. 75 Endovascular repair may be used to seal the entry between the true and false lumen to decrease the blood flow through the false lumen and promote stabilization through thrombosis in the descending aorta. Additionally, in patients with DeBakey-I dissections, antegrade thoracic stent grafting can be utilized to obliterate the false lumen in up to 80% of patients, as described by Pochettino et al. 38 Endovascular interventions have been shown to be well tolerated with antegrade stent graft deployment being a safe method to obliterate the thoracic false lumen. This type of “elephant trunk” thoracic stent-grafting provides equivocal short-term results compared with standard, open repair and lowers morbidity and mortality in the long-term.

Given the increased risk of aortic enlargement, reentry, and chronic dissection, it is indicated to monitor patients with a patent false lumen more closely to assess lumen status. Furthermore, select patient groups may benefit from endovascular repair or supplemental stent grafting.

### Outcomes in the Current Era

The culmination of previously discussed controversies is an analysis of the clinical outcomes following repair over time. A previous study described a decline in operative mortality over the period from 1963 to 1992, with findings summarized in Table 4 . 76 However, with broad understanding of disease and advances in surgical technique, how has the survival trend changed in the current era?

To address this question, Conway et al 77 compared 111 patients who underwent repair between 2000 and 2005 with 140 patients who underwent repair between 2006 and 2010. This study demonstrated that operative mortality was significantly influenced by surgical era, with a 24% operative mortality rate in patients treated between 2000 and 2005 compared with 12% in patients treated between 2006 and 2010 ( *p* = 0.013). Independent predictors of operative mortality as described by multivariate logistic regression included hemodynamic instability (OR = 17.8; 95% CI: 0.05–0.35; *p* < 0.001), CPB time > 200 minutes (OR = 9.5, 95% CI: 0.14–0.64; *p* = 0.002), and earlier date of surgery (OR = 5.8; 95% CI: 1.18, 5.14; *p* = 0.016). Additionally, actuarial 5-year survival was significantly worse for patients treated earlier (64% for 2000–2005, 77% for 2006–2010, *p* < 0.001). 77 These findings demonstrate that the early clinical outcomes of repair of Type A aortic dissection have improved over time.

## Conclusions

A long history of research on AAAD repair demonstrates significant progress. However, the literature also exposes areas of controversy. Current literature review indicates that use of moderate hypothermia and antegrade cerebral perfusion is a safe alternative to deep hypothermia. Furthermore, axillary cannulation maybe used instead of femoral artery in hemodynamically stable patients who don't require emergent institution of cardiopulmonary bypass. Challenges still include the treatment of the hemodynamically unstable patients, as well as those with malperfusion. With regard to the technical aspects of repair, preserving the aortic root whenever possible, and performing the distal anastomosis with the open distal technique rather than with the clamp on is the preferred approach. In patients with a patent false lumen, close monitoring is indicated. As demonstrated by the literature, significant improvement of early and late mortality over the past years has occurred in patients presenting with AAAD.
